# Evaluation of canine teeth crown reduction technique in macaques

**DOI:** 10.1186/s42826-020-00051-3

**Published:** 2020-06-05

**Authors:** Su-Mi Kim, Jong-Min Kim

**Affiliations:** 1 Seoul National University Dental Hospital, 103 Daehak-ro Jongno-gu, Seoul, 110-799 South Korea; 2 Xenotransplantation Research Center, Seoul, South Korea; 3 Institute of Endemic Diseases, Seoul, South Korea; 4 Cancer Research Institute, Seoul National University College of Medicine, 103 Daehak-ro Jongno-gu, Seoul, 110-799 South Korea

**Keywords:** Canine teeth reduction, Macaque, Personnel protection

## Abstract

**Background:**

The reduction of canine teeth in adult males in whom permanent canine teeth eruption occurs should be considered due to the safety of humans or other monkeys. The objective of this study was to evaluate the complications of canine teeth reduction in macaques. Case presentation: Canine teeth reductions were performed in 8 rhesus and 2 cynomolgus macaques. Complications related to canine teeth reduction were evaluated at one to three week intervals during the experimental period by gross examination, CBC, and monitoring of appetite. One monkey showed a fistula due to periapical abscess and neutrophilia more than 2 years after canine teeth reduction, and extraction of the diseased canine tooth was performed; the other 9 monkeys showed no complications during the observation period. This report shows the effectiveness of canine teeth reduction with few complications.

**Conclusion:**

Canine teeth reduction in male macaques is an acceptable procedure for reducing the severity of injury to humans or other monkeys.

## Background

Permanent canine teeth eruption occurs at approximately 43 months old and approximately 41 months old in the maxilla and mandible, respectively, in rhesus monkeys (*Macaca mulatta*) and cynomolgus monkeys (*Macaca fascicularis*) [1]. Complete maturation of the canine teeth (closed apex) has been confirmed by the radiographic assessment of monkeys between 6 and 7 years old [2]. At this time, male canine teeth become long, sharp, and powerful for survival in the wild due to the eruption of the canine teeth to complete maturation. As canine teeth become sharper after eruption, canine teeth reduction has been used for personnel protection [2, 3]. For the safety of researchers, canine teeth reduction can be conducted, and complications can be evaluated until the end of experiments in macaques [4]. The objective of this report was to evaluate canine teeth reduction in terms of complications in macaques.

## Case presentation

**Fig. 1:**
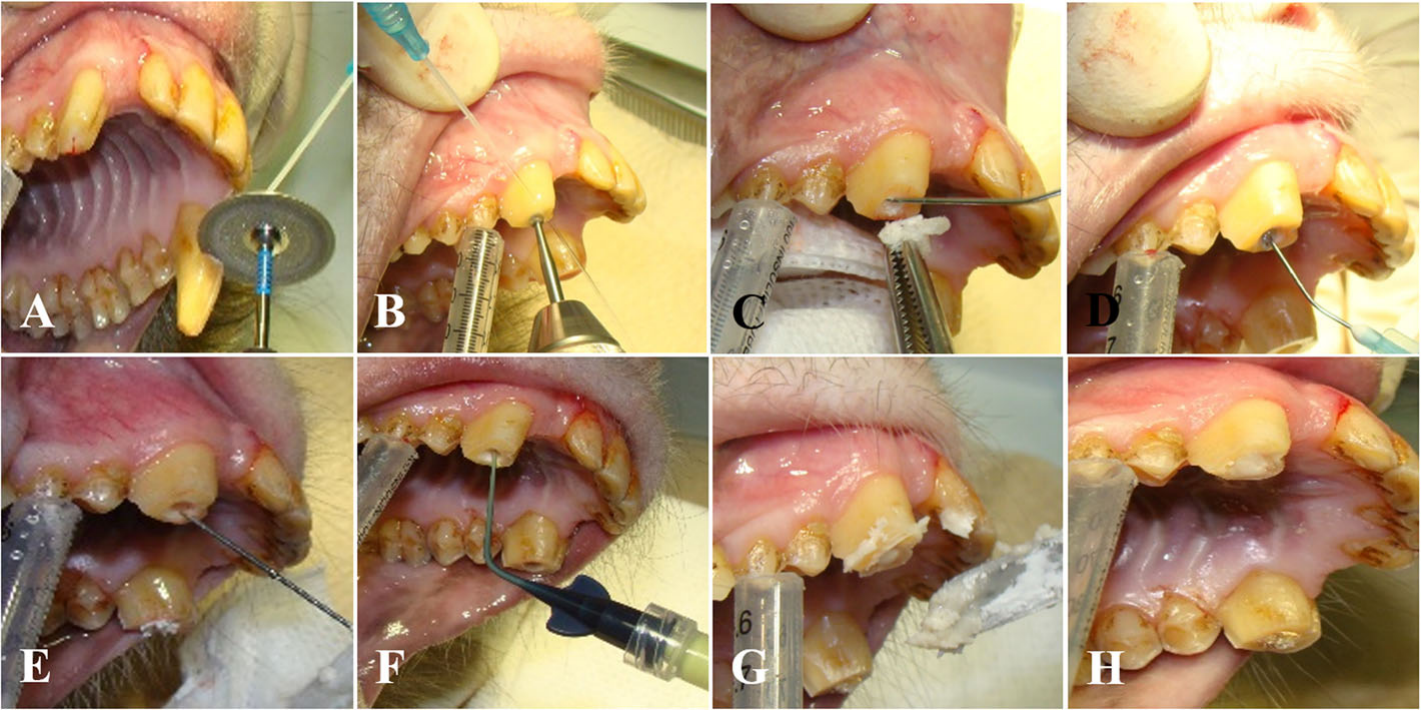
Images of the canine teeth crown reduction technique. **a** Left maxillary canine tooth is cut to the level of the incisor teeth using a disc bur. **b** A 1.5-mm diameter bur removes the pulp and dentin with saline irrigation to prevent thermal injury, and the removed pulp cavity has a larger diameter at the base compared to the cutting surface. **c** A cotton pellet soaked with a hemostatic agent (dental formocresol; AGSA JAPAN CO., Osaka, Japan) is used to control the bleeding induced by pulpotomy. **d** A phosphoric acid etchant (CharmEtch 35 HV; DentKist Inc. Gunpo, Gyeonggi-do, Korea) is applied for 20 s for strong adhesion to the filling material. **e** The cavity is washed with a 5.25% sodium hypochlorite solution with antimicrobial effects and then dried gently with air. **f** The cavity is filled with calcium hydroxide/iodoform paste (Vitapex, Neo Dental Clinical Co., Tokyo, Japan). **g** The cavity is filled with glass-ionomer restorative cement (Fuji IX GP; GC Corporation, Tokyo, Japan) according to the manufacturer’s protocol. **h** Complete image of canine tooth crown reduction

**Fig. 2:**
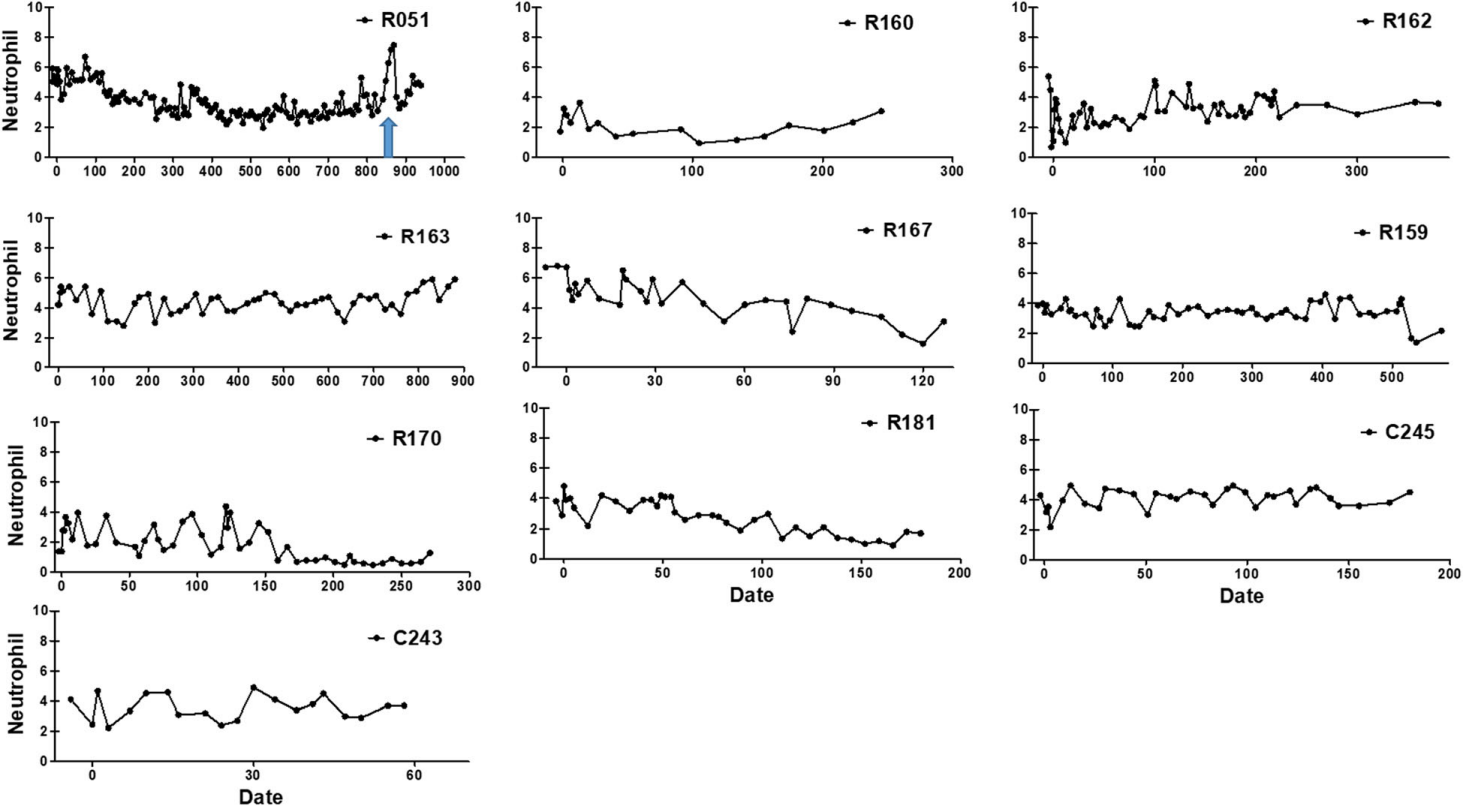
Images of level of neutrophil during observation periods after canine teeth crown reduction. There are no neutrophilia except R051. The neutrophilia (arrow) with fistula on the attached gingiva are observed around D870 in R051, but it returns to the normal range after left maxillary canine tooth extraction

**Fig. 3:**
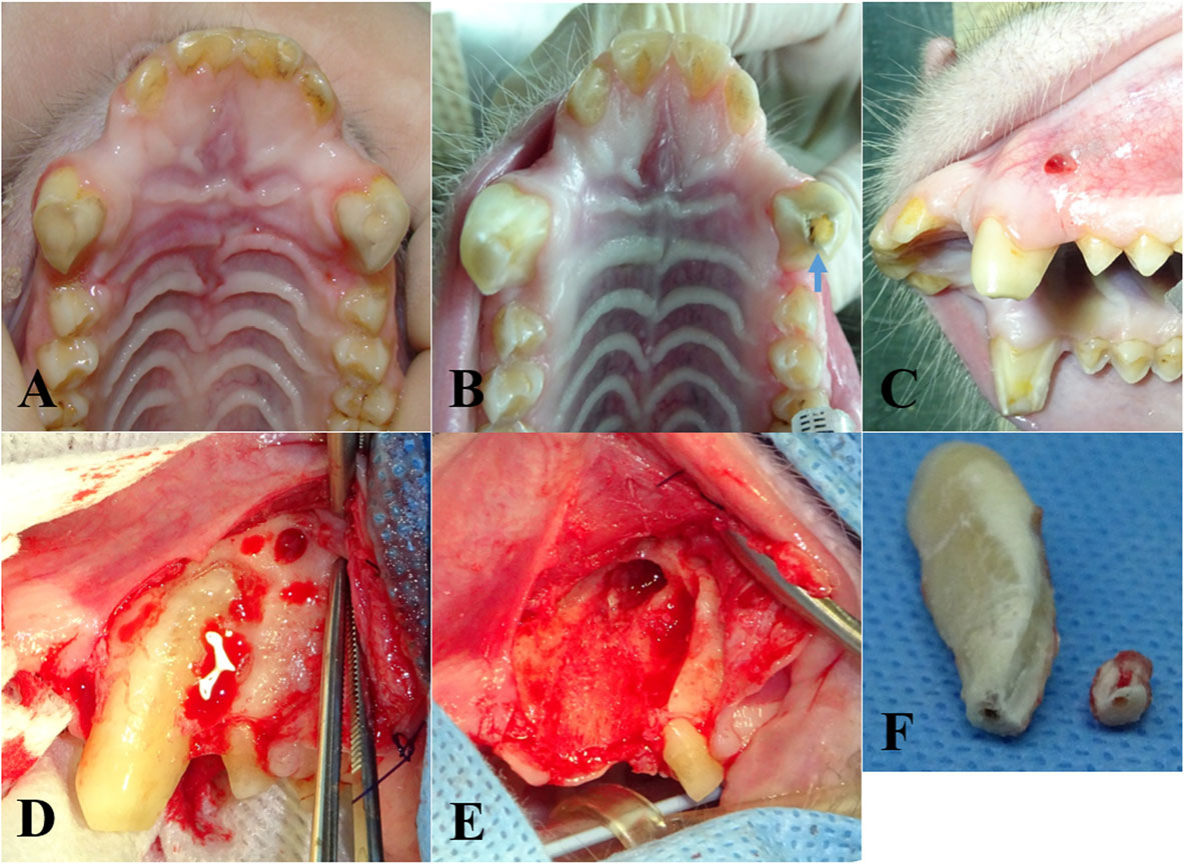
Images of a fistula due to periapical abscess and alveolectomy of the left canine tooth. **a** Image after canine teeth crown reduction. **b**: The glass-ionomer restorative cement (arrow) fell out into the cavity of the left canine tooth. **c**: Fistula is observed on the attached gingiva. Images **b** & **c** were photographed 2 years and 6 months after canine teeth crown reduction. **d** Buccal alveolectomy is performed before left maxillary canine tooth extraction. **f** Image after buccal alveolectomy and canine tooth extraction. **f** Extracted canine tooth that was broken during tooth extraction

Four- to 9-year-old male macaques (8; rhesus, 2; cynomolgus) were included in this experiment (Table 1). The animal experiments were approved by the Institutional Animal Care and Use Committee (IACUC) of the Biomedical Research Institute at the Seoul National University Hospital (an AAALAC accredited facility); IACUC number: 14–0034, 15–0297, and 16–0192). Canine teeth reduction was performed with a sterile procedure that reduces the height of the lower canine crowns by exposing the pulp and removing some of the pulp (partial coronal pulpectomy) as well as the placement of direct pulp capping (Fig. 1). Briefly, canine tooth is cut to the level of the incisor teeth using a disc bur. A 1.5-mm diameter bur removes the pulp and dentin with saline irrigation to prevent thermal injury, and the removed pulp cavity has a larger diameter at the base compared to the cutting surface. A cotton pellet soaked with a hemostatic agent (dental formocresol; AGSA JAPAN CO., Osaka, Japan) is used to control the bleeding induced by pulpotomy. A phosphoric acid etchant (CharmEtch 35 HV; DentKist Inc. Gunpo, Gyeonggi-do, Korea) is applied for 20 s for strong adhesion to the filling material. The cavity is washed with a 5.25% sodium hypochlorite solution with antimicrobial effects and then dried gently with air. The cavity is filled with calcium hydroxide/iodoform paste (Vitapex, Neo Dental Clinical Co., Tokyo, Japan). The cavity is filled with glass-ionomer restorative cement (Fuji IX GP; GC Corporation, Tokyo, Japan) according to the manufacturer’s protocol. From 2 months to 2 years and 5 months until the end of the experiment, complications related to canine teeth reduction were evaluated at 1- or 3-week intervals under sedation (medetomidine 10 mcg/kg + ketamine 5 mg/kg, intramuscular injection) in terms of CBC, monitoring of appetite, gingival edema or erythematous, intactness of the capping material and the presence of fistula due to periapical abscess. All macaques recovered well during the examination period after canine teeth reduction and returned to normal conditions without complications except R051. The level of neutrophil increased around D870 in R051 (Fig. 2). The complication of dental abscess could be evaluated by CBC and if there were bacterial infections, there should be an increase in neutrophil count [5]. The glass-ionomer restorative had fallen out from left the canine tooth, and a fistula with pus on the buccal gingiva above the left maxilla canine tooth was found in R051 at 2 years and 5 months after canine teeth reduction (Fig. 3; A~C). It is thought that the falling out of the capping material is caused by the biting cage rods for unknown reasons, perhaps habitually or under stressful conditions. Periapical abscess related to canine teeth reduction of the left maxilla canine tooth was diagnosed. Left maxilla canine tooth extraction was conducted via buccal alveolectomy (Fig. 3; D ~ F). The incised gingiva were closed with a routine method. The wound healed without complications, and R051 returned to a normal status. An antibiotic (Cefazolin 20 mg/kg, bid) and an analgesic (meloxicam 0.1 mg/kg, sid) were injected intramuscularly for 3 days after canine teeth reduction or after left maxillar canine tooth extraction.

**Table 1:** Observation periods after canine teeth crown reduction in macaques

ID of macaques	Age at the time of canine teeth crown reduction	Observation periods after canine teeth crown reduction
R051	9y 11 m	2y 5 m
R160	8y 1 m	8 m
R162	8y	1y 1 m
R163	8y 6 m	2y 5 m
R167	8y 5 m	4 m
R159	7y 3 m	1y 7 m
R170	4y 11 m	9 m
R181	9y 5 m	6 m
C245	5y 2 m	6 m
C243	4y 7 m	2 m

*R*: rhesus monkey, *C*: cynomolgus monkey

## Discussion and conclusions

This study shows comparable results, such as the safety of researchers and few complications (R051) after canine teeth reduction, although there was a relatively short examination period. Usually, tooth abscess occurs 2 or 3 years after canine teeth reduction. Only 2 monkeys among 12 were observed for more than 2 years after canine teeth reduction because the other 10 monkeys’ experimental periods were short. However, in terms of safety concerns for researchers, some researchers were previously bitten and had puncture wounds on the skins in front of the chest and hand by male macaques even though personnel protective equipment was applied. After obtaining the puncture wounds, the researchers had to take a prophylactic anti-herpes viral agent to prevent B virus infection [6] in my institute, which is the most dangerous zoonosis in monkeys. After canine teeth reduction was administered, the researcher had no puncture wounds due to being bitten by the male macaques. It is thought that the advantages outweigh the disadvantages in lieu of various aspects.

A limitation of this study is that evaluation by dental radiography was not performed due to the lack of dental X-ray machines in my institute. Dental radiography is the gold standard for the evaluation of endodontic treatment, especially periapical abscesses [7]. Although dental radiography was not performed, a fistula with pus and unviable pulp on the extracted canine tooth with missing capping material was used to make the diagnosis of periapical abscess.

From the perspective of animal welfare, the practice of canine tooth reduction for the purpose of providing personnel safety has been soundly opposed by the American Veterinary Medical Association (AVMA) and Association of Primate Veterinarians (APV); therefore, this procedure is rarely performed in the United States of America when required for medical treatment or scientific research approved by an institutional animal care and use committee (personal communication). However, it is thought that canine teeth reduction and the extraction of canine tooth by alveolectomy with periapical abscess as a complication of canine teeth reduction may have value to institutions outside of the United States of America. The reduction of canine teeth in male monkeys is an acceptable procedure for reducing the severity of injury to humans or other monkeys.

## Data Availability

Not applicable.

## Abbreviations

AAALAC: Association for Assessment and Accreditation of Laboratory Animal Care
APV: Association of Primate Veterinarians
AVMA: American Veterinary Medical Association
IACUC: Institutional Animal Care and Use Committee
